# Broad Spectrum Polyphenol Supplementation from Tart Cherry Extract on Markers of Recovery from Intense Resistance Exercise

**DOI:** 10.1186/s12970-021-00449-x

**Published:** 2021-06-14

**Authors:** D. R. Hooper, T. Orange, M. T. Gruber, A. A. Darakjian, K. L. Conway, H. A. Hausenblas

**Affiliations:** 1 Center for Health and Human Performance, Jacksonville University, Jacksonville, USA; 2 Department of Kinesiology, Brooks Rehabilitation College of Healthcare Sciences, Health Sciences Complex, Jacksonville University, 2800 University Boulevard North, Jacksonville, FL 32211 USA; 3 Department of Biology and Marine Science, Jacksonville University, Jacksonville, USA

**Keywords:** Tart cherry, antioxidants, polyphenols, recovery, muscle damage, cardiac damage

## Abstract

**Background:**

Tart cherry supplementation has been shown to enhance recovery from strenuous exercise due to its antioxidant properties. The majority of these studies used tart cherry juice, with a significant calorie content. The primary purpose of this study was to assess whether powdered tart cherry extract with minimal calorie content reduces oxidative stress and enhances recovery following intense resistance exercise.

**Methods:**

Thirteen men (mean age: 26.2 ± 5.3 years; height: 184.3 ± 8.2 cm; weight: 92.9 ± 15.6 kg) performed a demanding resistance exercise protocol consisting of 6 sets of 10 repetitions of barbell back squat with 80% 1RM. The protocol was performed once following 7 days of 500 mg of tart cherry extract and once following placebo. Serum protein carbonyl (PC) content, creatine kinase activity (CK) and creatine kinase myocardial band content (CK-MB) were used to assess oxidative stress, skeletal and cardiac muscle damage respectively. Muscle soreness was assessed by visual analog scale. Physical performance was measured by countermovement jump power and handgrip dynamometer strength.

**Results:**

There was a significant increase in PC in the placebo (PL) condition when compared to the Tart Cherry (TC) condition at Immediate Post (IP) (PL: 0.4 ± 0.3 vs. TC: − 0.4 ± 0.2 nmol∙mg^− 1^; *p* < 0.001), 1 h (PL: 0.3 ± 0.3 vs. TC: − 0.7 ± 0.3 nmol∙mg^− 1^; p < 0.001) and 24 h (PL: 0.1 ± 0.4 vs. TC: − 0.3 ± 0.5 nmol∙mg^− 1^; *p* = 0.010). There was a significant increase in CK activity in PL when compared to the TC at IP (PL: 491.1 ± 280 vs. TC: 296.3 ± 178 U∙L^− 1^; *p* = 0.008) and 3 h (PL: − 87 ± 123 vs. TC: 43.1 ± 105.3 U∙L^− 1^; *p* = 0.006). There was a significant (*p* = 0.003) increase in CKMB concentration in PL when compared to the TC (PL: 21.6 ± 12.4 vs. TC: − 0.3 ± 11.8 ng∙ml^− 1^; *p* = 0.006) at 1 h post. There was a significant increase in handgrip strength in TC when compared to PL (PL: − 2 ± 5.1 vs. TC: 1.7 ± 3 kg; *p* = 0.017) at 24 h post.

**Conclusions:**

This study demonstrated that tart cherry extract reduced oxidative stress and markers of muscle and cardiac damage following intense resistance exercise. This occurred along with a prevention of the decrease in handgrip strength seen following the intense exercise protocol, indicating a potential reduction in central fatigue. These benefits were seen with minimal energy intake.

## Background

Resistance exercise is a powerful stimulus to induce gains in strength and hypertrophy [1]. Although such gains can lead to enhanced athletic performance and may reduce the demands of activities of daily living, paradoxically resistance exercise increases markers of oxidative stress [2, 3], skeletal [4] and cardiac [5] muscle damage, and transiently reduces physical performance [6]. These responses to exercise are not necessarily negative, but rather are a necessary part of the adaptation process, providing the level of stress does not exceed the ability of the body to recover.

However, if the stress caused by exercise is too great and the body is unable to recover adequately, long term reductions in performance level can occur, such as in non-functional overreaching, or overtraining [7], or in much more severe cases, can be life threatening in the form of rhabdomyolysis [8]. Supplementation with plant compounds has been suggested to be able to reduce inflammation, oxidative stress and subsequent skeletal and cardiac muscle damage associated with intense exercise, thereby enhancing recovery and helping to stimulate optimal adaptation.

One such group of plant compounds are polyphenols, which are naturally high in fruits and vegetables. Although polyphenols themselves are radical scavengers, they exist in such low concentrations in human blood that their ability to reduce markers of oxidative stress are more likely due to their capacity to enhance endogenous anti-inflammatory and antioxidant mechanisms through the nuclear factor erythroid 2-related factor 2 (Nrf2) pathway than by directly scavenging themselves [9].

One source of polyphenols that has received considerable attention in this area is tart cherries [3, 6, 10–16]. Following various forms of intense physical activity, such as resistance exercise [3, 6, 13], long distance running [14, 16], cycling [11, 12], repeated sprints [10], or sport [15], consumption of tart cherries reduces markers of oxidative stress [3, 11, 16], muscle soreness [10, 13, 14] and attenuates reductions in physical performance [3, 10, 12, 13, 16]. These results are not unequivocal, however, likely due to differences in dosing and damage protocol [9]. Complicating the interpretation further, the enhanced recovery is not always seen as some studies failed to show either an increase in muscle damage [11], or a reduction in performance [15] from the chosen protocol, giving the supplement no damage/performance measure to recover from.

While there are many ways to assess oxidative stress, one particular method that has been used to demonstrate the effectiveness of a polyphenol supplement to reduce its effects following exercise has been by a decrease in serum protein carbonyls (PC), a marker of oxidative damage [2]. For example, Bowtell et al. [3] saw a significant reduction in PC following 10 sets of 10 knee extensions at 80% 1RM following a Montmorency cherry supplementation when compared to an isoenergetic fruit concentrate. Also, Chang et al. [17] demonstrated a reduction in PC following 1 h of treadmill running with the use of a polyphenol supplementation in the form of purple sweet potato when compared to control.

One variable not measured in prior polyphenol research, but has been assessed in other antioxidant literature, is creatine kinase myocardial band (CK-MB), a marker typically associated with cardiac damage [5] that has recently been proposed as a marker specifically for type I muscle fiber damage [18]. An increase in CK-MB is a typical response to exercise and is associated with the structural disruption of muscle fibers and the leaking of the contents on these fibers into the plasma [5]. Strenuous exercise, such as long distance running [18], professional soccer [19] and also resistance exercise [5] significantly increases CK-MB. These increases have been attenuated by other antioxidant supplements, such as Vitamin E under hypoxic conditions [20] and the drug Allopurinol [19], and thus antioxidants could potentially enhance recovery from strenuous exercise in the context of CK-MB.

While it stands to reason that aerobic activity, when compared with anaerobic activity, would be the more likely beneficiary of an antioxidant supplementation due to its reliance on oxidative metabolism, oxidative stress has been demonstrated following resistance training and there are alternative ways that oxidative stress can impact recovery. For example, many studies have assessed the impact of polyphenol supplementation on skeletal muscle damage in the form of changes in serum creatine kinase (CK) and/or muscle soreness. The prevailing rationale for such analyses is that while mechanical stress is a major contributor to skeletal muscle damage, an increase in reactive oxygen species could cause oxidative modifications of skeletal muscle proteins. Thus, an antioxidant could potentially reduce biochemical damage [9]. This rationale has received equivocal support, with some studies showing a benefit of polyphenol supplementation [6, 14, 21–23] and others not [3, 11, 12, 16, 24–26]. In terms of the studies that utilized resistance exercise, outside of Levers et al. [6], all utilized unilateral strength exercise or parallel group design, which are susceptible to high inter-individual differences and could explain the lack of agreement in the literature [9].

While changes in oxidative stress and/or markers of muscle damage would be promising to demonstrate whether polyphenol supplementation is efficacious, it would be important to demonstrate a measureable improvement in physical performance level resulting from that reduced oxidative stress. While the majority of research has demonstrated some form of enhanced performance level during the recovery process as a result of polyphenol supplementation, such as maximal voluntary contraction [3, 12, 24, 27], isokinetic performance [21, 25] or time trial performance [10], some studies have failed to show performance benefits [6, 15, 26]. However, it is important to note that neither Peschek et al. [26] nor McCormick et al. [15] successfully reduced their performance measures with their exercise protocol, thus providing no performance decrement for the supplements to attenuate. The final study that failed to show performance improvement by Levers et al. [6] saw a significant reduction in performance at only 1 of the 4 time points that were measured, again providing the supplement little opportunity to demonstrate effectiveness. Overall, it appears that when performance level is reduced by strenuous exercise, the overwhelming majority of studies do demonstrate an enhanced recovery of performance with polyphenol supplementation over placebo.

Even when reductions in muscle damage and soreness are observed in conjunction with enhanced recovery of physical performance measures with the use of a nutritional supplement, an individual would still need consider the benefits of the supplement over the consumption of the additional energy and whether that would fit with their intended dietary intake. On this note, the overwhelming majority of the literature pertaining to the effects of cherry derived antioxidants on muscle damage and performance have used fruit juices, containing at least 100 kcal of energy, with all using multiple doses (2–3 per day) over many days (up to 10 days) [3, 11–16]. Very few studies have assessed the effects of the antioxidant containing extracts in the absence of any macronutrients, although they have suggested potential benefits [6, 28]. The use of tart cherry supplementation in the absence of added macronutrients has involved consuming the supplement in powder form [6, 28], as opposed to juice form containing significant amounts of sugar. Interestingly, these powder studies used considerably lower doses of polyphenols (500 mg vs. > 1000 mg) but still demonstrated ergogenic effects [6, 28].

The primary purpose of this study, using a randomized crossover design, was to assess whether polyphenol supplementation in the form of a powdered tart cherry extract reduces oxidative stress, skeletal and cardiac muscle damage, and muscle soreness following intense bilateral resistance exercise. The secondary purpose was to determine whether the recovery of physical performance was enhanced as a result. We hypothesized the polyphenol supplementation would result in reduced muscle damage and soreness and the recovery would be enhanced following intense resistance exercise.

## Methods

### Subject Background and Preparation

Thirteen men (mean age: 26.2 ± 5.3 years; height: 184.3 ± 8.2 cm; weight: 92.9 ± 15.6 kg; barbell back squat 1RM: 146.8 ± 30.6 kg) with a minimum of 6 months of prior experience in the barbell back squat completed the study. All subjects provided written informed consent after approval for the study was granted by the Institutional Review Board at Jacksonville University.

### Experimental Overview

**Fig. 1:**
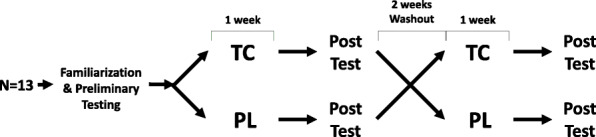
Study Design. *TC* Tart Cherry Supplementation, *PL* Placebo

The study utilized a randomized, cross-over, counter-balanced, placebo controlled design, as illustrated in Fig. 1. Subjects initially attended familiarization and preliminary testing. During this visit, subjects were introduced to the facility and walked through the basic procedures, including muscle soreness assessment, the warm-up protocol, and the vertical jump and handgrip dynamometer procedures, followed by a 1-repetition maximum assessment on the barbell back squat.

For muscle soreness, subjects were asked to report the amount of muscle soreness that they were currently perceiving in their upper legs. While the subject was seated with legs elevated, a researcher palpated with both hands the upper, middle and lower quadriceps, applying firm pressure with the thumb and forefinger for approximately 1 s. The subject then marked a line on a 10 cm scale that corresponded to their muscular soreness. The warm-up consisted of 5 min on a Monark exercise bike (Vansbro, Sweden) keeping a constant speed of 60–80 rpm. Following the exercise bike, subjects performed a range of dynamic stretching exercises, including bodyweight squats, lunges, lateral lunges, knee hugs, quad pulls and straight leg marches. Each dynamic stretch was performed for 10 repetitions.

Vertical jump was used to assess the impact of the muscle damage protocol on muscle performance and to identify if the supplement could enhance recovery of this measure. For the vertical jump assessment, subjects were instructed to stand on a NeuroCom Balance Manager forceplate (Natus, Pleasanton, CA) with hands on hips. Subjects were instructed to perform 3 consecutive vertical jumps, as high as possible with minimal time between jumps. Jumps were later analyzed for power using the equation described by Sayers et al. [29].

As grip strength has been previously demonstrated to be associated with central fatigue [30, 31], it was used as a marker of recovery. Grip strength of the dominant hand was measured with a grip strength dynamometer (Hydraulic Hand Dynamometer, North Coast Medical Inc., Morgan Hill, USA). The highest score of three attempts was used in the analyses.

For the 1-Repetition Maximum Testing (1RM), subjects performed squats for 8–10 repetitions at ~ 50% of estimated 1RM followed by another set of 2–5 repetitions at ~ 85% of 1RM. Subsequently, 3–5 maximal trials were used to determine the individual’s 1RM, each separated by 3 min of rest. Necessary squat depth was standardized as the upper thigh reaching at minimum a position that was parallel to the ground. The highest load lifted for 1 repetition with correct technique was considered their 1RM.

### Supplementation

Following the familiarization visit, subjects took the supplement daily for 1 week prior to the acute resistance exercise protocol (AREP). A large body of evidence has shown that supplementing with polyphenols daily for 3 or more days prior to and following exercise will enhance recovery [9]. The experimental supplement was a specialized, proprietary broad spectrum Tart Cherry Extract Powder (NordicCherry®) manufactured by Specnova, LLC. The supplement contained a broad spectrum of polyphenols with total polyphenols 5–6% w/w tested via F-C assay. The subjects received a dose of 500 mg in capsule form containing 1.3 kcal, 0.3 g carbohydrate, 0 g fat and 0.008 g protein. For the placebo, the subjects received rice flour. Both the experimental and placebo supplements were contained in opaque capsules, size 00, thus blinding the subjects to their supplement. Participants were provided with the exact number of pills required to be taken from the time they received the supplement until their next visit to the laboratory, and asked to return the empty container. No side effects related to the supplement of any kind were reported during the study. The supplement and placebo were prepared by Specnova, LLC.

### Acute Resistance Exercise Protocol (AREP)

Subjects arrived at the Exercise Physiology Laboratory following an overnight fast. Subjects were encouraged to drink 2 cups of water the evening prior and 2 cups of water the morning of the visit to ensure adequate hydration. Before, immediately following, 1 h and 3 h after the protocol, blood was drawn from an antecubital vein into a serum vacutainer.

Once the baseline blood sample was taken, the subject began the warm up, as described earlier. Following the warm up, the barbell was loaded with a weight corresponding to 80% of their predetermined 1-repetition maximum. The subject then aimed to perform 10 repetitions of the barbell back squat for 6 sets interspersed with 2 min of rest. If the subject was unable to complete 10 repetitions for a given set, the subsequent set was then performed with 75% 1RM. This procedure of reducing the load 5% 1RM for each set where the subject fails to complete 10 repetitions was repeated for all sets. When the AREP was completed on the second occasion, the exact protocol that the subject followed (in terms of weight lifted and repetition number for each set) during the first AREP was repeated. Following completion of the AREP, an immediate post-blood was taken. Subsequent blood draws also took place 1 h and 3 h following the protocol.

### Recovery Visits

At 24 and 48 h after the AREP, a single blood draw was conducted followed by a measurement of handgrip strength and jump power. The blood draw took place following a 10-min seated rest period. During the 10-min rest period, muscular soreness was assessed with a 10 cm visual analog scale. Handgrip strength and vertical jump power were then assessed with the procedures previously described.

### 2 Week Washout

Following the first cycle of testing, the subjects underwent a 2 week washout period. During this time no supplement was consumed and the subjects were instructed to resume their normal diet. Following the two week washout, the subjects completed the entire data collection process a second time (7 day loading period, AREP and recovery visits) with the alternate condition (i.e., supplement or placebo).

### Serum Analyses

The initial blood draw prior to the AREP took place following 10 min of seated rest to ensure a true resting blood draw. Other than at the Immediate Post (IP) time point, all draws were conducted following a minimum of 10 min of seated rest. Blood was spun at 1500 × g for 10 min at room temperature and serum was aliquoted and stored at −80 °C until subsequent analyses. Serum was analyzed in bulk at the completion of the study in our laboratory. Protein carbonyls (PC) were quantified by an Enzyme-Linked Immunosorbent Assay (ELISA) kit (OxiSelect™ Protein Carbonyl ELISA Kit, Cell Biolabs, Inc., San Diego, CA). Serum protein concentration was determined for each sample using the Bradford Protein Assay (Bio Basic, Amherst, NY) prior to running the ELISA and samples were run in triplicate per manufacturer’s protocol.

Creatine kinase (CK) activity was assessed using a colorimetric assay (Creatine Kinase Activity Assay Kit, Abcam, Cambridge, MA). Reference values for CK are 38–174 U∙L^− 1^. Serum samples were diluted 1:5 and readings were taken over time for 10 min. Sample preparation and analysis were performed per manufacturer’s protocol.

Creatine kinase isoenzyme MB was assayed by ELISA per manufacturer’s protocol (Human CKMB (CKM/CKB) ELISA Kit, Thermo Scientific, Frederick, MD). Reference values for CKMB are 0–10 ng∙ml^− 1^.

### Statistical Analyses

All data are presented as means along with standard deviation. Data were confirmed to demonstrate a normal distribution according to Shapiro-Wilk. Data points greater than 2 standard deviations from the mean were removed as outliers. Less than 2.5% of the data were removed as outliers. If the baseline value for a subject was not attainable for a certain variable, the subject was removed from analysis for that variable. Missing data was replaced with the mean delta for that time point. A 2-way repeated measures ANOVA was conducted for PC, CK and CKMB. A 2-way repeated measures ANOVA was conducted for muscle soreness, jump power and handgrip dynamometer measurements. The Mauchley sphericity test was conducted to confirm homogeneity of variance; if this assumption was violated, the Greenhouse-Geisser adjustment was performed If statistical significance was observed, post-hoc analyses were conducted in the form of paired *t*-tests on the change from baseline values at each time point, with a Bonferroni correction factor to account for alpha inflation. All statistics were run using SPSS Statistical Software, Version 24 (IBM Corporation, Armonk, NY, USA).

## Results

Raw measures of oxidative stress, muscle damage and physical performance parameters are illustrated in Table 1.

**Table 1:** Raw measures of oxidative stress, muscle damage and physical performance parameters for 13 men following a heavy resistance exercise protocol after supplementing with placebo and tart cherry supplementation

		Time Point					
	Group	BL	IP	1 h	3 h	24 h	48 h
PC (nmol∙mg^− 1^)	TC	2.43 (0.73)	2.01 (0.65)	1.73 (0.76)	2.14 (0.88)	2.09 (0.58)	2.23 (0.66)
	P	2.03 (0.52)	2.5 (0.7)	2.44 (0.57)	2.34 (0.64)	2.23 (0.56)	2.37 (0.67)
CK (U∙l^−1^)	TC	254 (106)	602 (253)	320 (126)	297 (110)	299 (89)	235 (95)
	P	322 (131)	813 (256)	343 (148)	269 (138)	354 (111)	305 (139)
CKMB (ng∙ml^−1^)	TC	22 (11)	20 (5)	21 (7)	20 (6)	23 (11)	20 (10)
	P	21 (9)	24 (11)	43 (18)	27 (10)	20 (7)	13 (9)
Soreness (cm)	TC	0.6 (0.6)				4.5 (2.2)	3.9 (1.8)
	P	0.7 (0.7)				5.7 (1.9)	5.9 (2.7)
Jump Power (W)	TC	3921 (869)				3699 (946)	3857 (887)
	P	3841 (878)				3897 (763)	3856 (803)
Handgrip (Kg)	TC	47 (6)				49 (6)	49 (6)
	P	50 (5)				48 (6)	50 (5)

*TC* Tart Cherry, *P* Placebo, *PC* Protein Carbonyls, *CK* Creatine Kinase, *BL* Baseline, *IP* Immediately Post, 1 h = 1 h post; 3 h = 3 h post; 24 h = 24 h post; 48 h = 48 h post

### Oxidative Stress

**Fig. 2:**
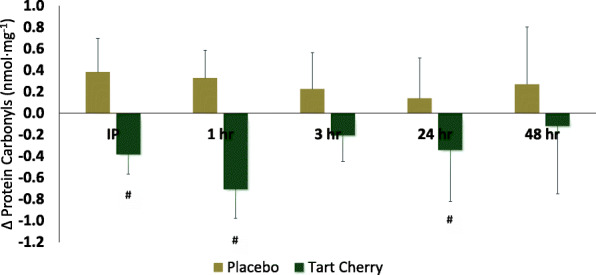
Changes in Protein Carbonyls (PC) from baseline for 13 men following a heavy resistance exercise protocol after supplementing with placebo and tart cherry supplementation. # = significantly (*p* ≤ 0.05) different from placebo at corresponding time point. *IP* Immediately Post; 1 h = 1 h post; 3 h = 3 h post; 24 = 24 h post; 48 = 48 h post

There was a significant main effect for supplement (*p* = 0.019), but not time (*p* = 0.520) and also a significant supplement*time interaction (*p* < 0.001). Figure 2 illustrates the changes in PC at each time point compared to baseline. There was a statistically significant increase in PC in the Placebo Condition when compared to the Tart Cherry Condition at IP (PL: 0.4 ± 0.3 vs. TC: − 0.4 ± 0.2 nmol∙mg^− 1^; *p* < 0.001), 1 h (PL: 0.3 ± 0.3 vs. TC: − 0.7 ± 0.3 nmol∙mg^− 1^; *p* < 0.001) and 24 (PL: 0.1 ± 0.4 vs. TC: − 0.3 ± 0.5 nmol∙mg^− 1^; *p* = 0.010).

### Muscle Damage

#### Creatine Kinase (CK) Activity

**Fig. 3:**
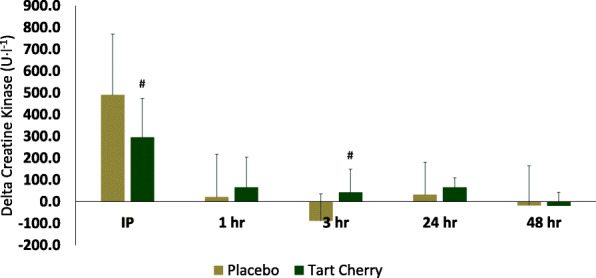
Changes in Creatine Kinase (CK) activity from baseline for 13 men following a heavy resistance exercise protocol after supplementing with placebo and tart cherry supplementation. # = significantly (*p* ≤ 0.05) different from placebo at corresponding time point. *IP* Immediately Post; 1 h = 1 h post; 3 h = 3 h post; 24 = 24 h post; 48 = 48 h post

There was a significant main effect for supplement (*p* = 0.036), time (*p* < 0.001) and also a significant supplement*time interaction (*p* = 0.003). Figure 3 illustrates the changes in CK activity at each time point compared to baseline. There was a statistically significant increase in CK activity in the Placebo Condition when compared to the Tart Cherry Condition at IP (PL: 491.1 ± 280 vs. TC: 296.3 ± 178 U∙L^− 1^; *p* = 0.008) and 3 h (PL: − 87 ± 123 vs. TC: 43.1 ± 105.3 U∙L^− 1^; *p* = 0.006).

#### Creatine Kinase Myocardial Band (CK-MB)

**Fig. 4:**
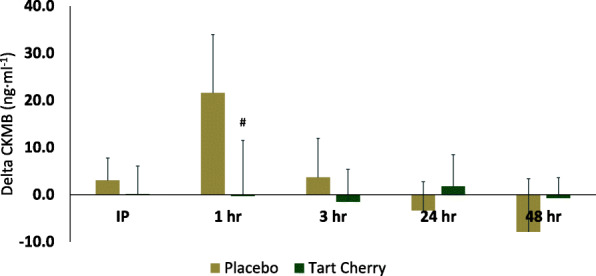
Changes in Creatine Kinase-MB (CKMB) concentrations from baseline for 13 men following a heavy resistance exercise protocol after supplementing with placebo and tart cherry supplementation. # = significantly (*p* ≤ 0.05) different from placebo at corresponding time point. IP = Immediately Post; 1 h = 1 h post; 3 h = 3 h post; 24 = 24 h post; 48 = 48 h post

There was a significant main effect for time (*p* < 0.001), and also a significant supplement*time interaction (*p* = 0.001), but no main effect for supplement (*p* = 0141). Figure 4 illustrates the changes in CKMB at each time point compared to baseline. There was a statistically significant (*p* = 0.003) increase in CKMB concentration in the Placebo Group when compared to the Tart Cherry group (PL: 21.6 ± 12.4 vs. TC: − 0.3 ± 11.8 ng∙ml^− 1^; p = 0.006) at 1 h post. There were no other statistically significant differences.

#### Muscle Soreness

**Fig. 5:**
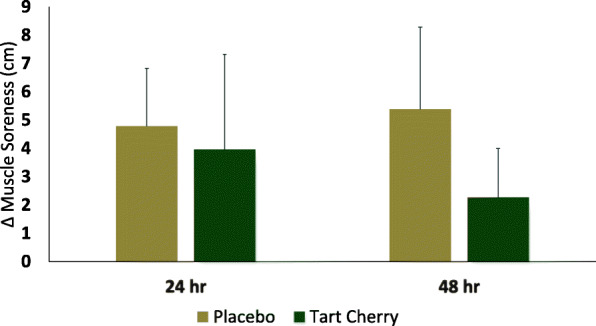
Changes in muscle soreness values from baseline for 13 men following a heavy resistance exercise protocol after supplementing with placebo and tart cherry supplementation. 24 h = 24 h post; 48 h = 48 h post

There was a significant main effect for time (*p* < 0.001), but a non-significant main effect for supplement (*p* = 0.136) and supplement*time interaction (*p* = 0.158). Muscle soreness changes at each time point when compared to baseline are displayed in Fig. 5. There were no other statistically significant differences.

### Physical Performance

There was a non-significant main effect for time (*p* = 0.396) and main effect for supplement (*p* = 0.145), but a significant supplement*time interaction (*p* = 0.041) for handgrip strength. Also, there was a non-significant main effect for time (*p* = 0.381) and main effect for supplement (*p* = 0.688), but a significant supplement*time interaction (*p* = 0.020) for jump power. Table 2 illustrates the changes in physical performance parameters at each time point when compared to baseline. There was a statistically significant increase in handgrip strength in the Tart Cherry group when compared to the Placebo Group (PL: − 2 ± 5.1 vs. TC: 1.7 ± 3 kg; *p* = 0.017) at 24 h post. There were no statistically significant differences, although jump height was trending (*p* = 0.073) towards significance (PL: − 7.3 ± 112.9 vs. TC: − 207.7 ± 291.5 W).

**Table 2:** Physical Performance. Changes in physical performance parameters for 13 men following a heavy resistance exercise protocol after supplementing with placebo and tart cherry supplementation

	Group	PL		TC		
Variable	Time Point	Mean	SD	Mean	SD	*p*
Jump Power (W)	∆ 24 h	−7.3	112.9	−207.7	291.5	0.073
	∆ 48 h	66.7	204.6	− 59.8	329.8	0.692
Handgrip Dynamometer (Kg)	∆ 24 h	−2.0	5.1	1.7#	3.0	0.034
	∆ 48 h	0.5	3.7	2.5	3.3	0.627

# = significantly (*P* ≤ 0.05) different from placebo at corresponding time point. 24 h = 24 h post; 48 h = 48 h post

## Discussion

The primary finding from this study is that the specialized, proprietary tart cherry supplementation was able to reduce oxidative stress and subsequently both skeletal and cardiac muscle damage, as well as reduce the attenuation of grip strength following an intense resistance exercise protocol when compared to placebo. This was achieved with extract containing minimal energy intake (1.3 kcal).

Paradoxically, while exercise is a powerful stimulus that can lead to positive changes in health and performance, it does induce an oxidative stress [32]. The typical increase in oxidative stress seen following intense exercise that was demonstrated in the placebo group was completely nullified by the supplement, as seen by negative PC delta scores at each time point (Fig. 2. These changes in PC have been demonstrated previously, both following resistance [3] and aerobic [17] exercise utilizing Montmorency cherry and purple sweet potato supplementation respectively. While Levers et al. [6] did not demonstrate changes in oxidative stress following tart cherry supplementation despite showing significant changes in muscle damage, the investigators did not address PC levels in that study. Thus, our data proposes PC may be a more sensitive indicator of changes in oxidative stress following polyphenol supplementation.

While an antioxidant supplement is unable to affect the mechanical stress placed on muscle tissue, by scavenging reactive oxygen species the supplement was able to reduce the amount of biochemical damage placed on the muscle proteins, as evidenced by significant decreases in both CK activity and CKMB. Although other polyphenol containing supplements such as blackcurrant juice [23] have demonstrated a reduction in skeletal muscle damage, this has not been previously shown in the tart cherry supplementation studies. In addition to skeletal muscle damage, this study assessed a marker of cardiac muscle damage [5], CKMB which was recently identified as a potential marker of type I muscle fiber damage [18]. While CKMB has been shown to decrease in the presence of other antioxidants, such as vitamin E [20] and Allopurinol [19], this study provides evidence that the compounds and fractions in tart cherries, including polyphenols, may have the same effect. CKMB may also be more sensitive to changes in muscle damage than CK activity following antioxidant supplementation, as several studies have failed to show differences in muscle damage following marathon running [16], cycling [12], intermittent sprints [10], or unilateral knee extension [3]. All of these prior studies utilized CK activity alone as their measure of muscle damage, rather than both CK activity and CKMB together, as we have done here. It is also noteworthy that although the supplement attenuated the increase in CK following the AREP, the increase CK was generally mild and would be categorized as low by prior studies [33, 34]. A more demanding AREP could have led to greater and more prolonged increases in CK, which could have given more opportunities for the supplement to demonstrate its effects.

Prior research has previously demonstrated reductions in muscle soreness [10, 13, 14] associated with antioxidant supplementation, however, these changes were not seen in this study (Fig. 5). Despite the reduction in oxidative stress and subsequent reduction in muscle damage, these were not manifested as perceivable changes in soreness to the subjects in this experiment. Other studies have also failed to demonstrate these changes in soreness even in the presence of attenuation in inflammation and oxidative damage [16], which ultimately illustrates the subjectivity and difficulty in quantifying soreness [9].

With regard to muscle damage, there may be a delicate balance. Muscle damage is a typical response to intense resistance exercise, following which the body recovers and adapts. However, if the recovery is inadequate, this can lead to a reduction in performance in the form of non-functional overreaching or overtraining [7]. Although this acute study was too short to induce non-functional overreaching, there was a significant attenuation of loss grip strength (Table 2). Loss of grip strength has previously been associated with central fatigue [30, 31], where reductions in grip strength were not seen from changes within the muscle, but rather from changes within the brain [31]. Thus, although speculative, it is possible that the fatigue induced by the AREP led to a reduction in the strength of the signal to produce force in the grip strength task in placebo group, but due to less oxidative stress and muscle damage in the TC group, this response did not occur and grip strength was maintained.

It is somewhat surprising that the supplement could have an effect on grip strength and not jump power considering the nature of the exercise protocol was the squat exercise. However, despite the intense lower body exercise, jump performance was not attenuated during the recovery visits, giving the supplement no performance decrements on which to improve. In a similar study, Levers et al. [6] utilized 70% 1RM in the barbell back squat as the muscle damage protocol, but did successfully induce reductions in performance in the form of maximal voluntary contractions 24 h following. Levers et al. [6], utilized 10 sets of squats, whereas this study used only 6 sets by comparison. This reduced volume is likely the reason for the failure for this study to induce performance decrements, particularly as the subjects in the two studies had remarkably similar strength levels (this study: 146.8 ± 30.6 vs. 142.2 ± 32.2 kg).

With regards to the limitations of this study, the exact amount of antioxidants consumed by the subjects is not known as there was while the subjects were encouraged to repeat their dietary intake during both arms of the study, this was not prescribed or monitored. In addition, the subjects were provided with the supplement and instructed to consume it, but they were not directly supervised ingesting the supplement. In terms of assessing oxidative stress, there are many ways this can be done, however this study only utilized one method in protein carbonyls. Other measures of oxidative stress could certainly have demonstrated different results. Finally, it appears the muscle damage protocol used in this study was mild in terms of either its intensity or volume as it produced only a modest increase in CK and failed to attenuate CMJ performance. In addition, the CK values reported may have been confounded by acute changes in plasma volume, which have been previously reported to occur with resistance training [35, 36]. If plasma volume changes had been measured in this study, the values could have been corrected accordingly.

## Conclusions

Polyphenol supplementation in the form of tart cherries has been shown to reduce oxidative stress and markers of muscle damage following intense resistance exercise. In addition, much like other plant compound supplements, polyphenols were shown to reduce CKMB, a marker of cardiac muscle damage. These declines in oxidative stress and muscle damage were associated with a significant strength benefit by preventing the decrease in strength seen following the intense exercise protocol, indicating a potential reduction in central fatigue. These benefits were seen with minimal energy intake. However, the protocol was not sufficient to cause reductions in power performance, and thus the supplement was unable to demonstrate reduced attenuations of performance as a result of the decreased damage. In the future, chronic studies are needed to show whether these reduced muscle damage markers and improved recovery of strength will translate to increases in long-term performance, or whether reducing the muscle damage markers could alter the necessary damage and recovery cycle needed for optimal adaptation.

## Data Availability

The datasets used and/or analysed during the current study are available from the corresponding author on reasonable request.

## Abbreviations

PC: protein carbonyl (PC) content,
CK: Creatine Kinase
CK-MB: Creatine Kinase Myocardial Band
TC: Tart Cherry
PL: Placebo
1RM: 1-Repetition Maximum
AREP: Acute Resistance Exercise Protocol
ELISA: Enzyme-Linked Immunosorbent Assay kit
